# The evolving definition of carcinogenic human papillomavirus

**DOI:** 10.1186/1750-9378-4-7

**Published:** 2009-05-11

**Authors:** Philip E Castle

**Affiliations:** 1 Division of Cancer Epidemiology and Genetics, National Cancer Institute, 6120 Executive Blvd. Room 5004, MSC 7234, Bethesda, MD 20892-7234, USA

## Abstract

Thirteen human papillomavirus (HPV) genotypes have been judged to be carcinogenic or probably carcinogenic, and the cause of virtually all cervical cancer worldwide. Other HPV genotypes could possibly be involved. Although the inclusion of possibly carcinogenic HPV genotypes may hurt test specificity, it may indirectly increase the reassurance following a negative HPV test (i.e. the negative predictive value of an HPV test for cervical precancer and cancer). The future of cervical cancer screening in low-resource setting, however, may include once-in-a-lifetime, low-cost and rapid HPV testing. However, the tradeoff of more false positives for greater reassurance may not be acceptable if the local infrastructure cannot manage the screen positives. Now is the time for the community of scientists, doctors, and public health advocates to use the data presented at the 100th International Agency for Research on Cancer monograph meeting to rationally decide the target HPV genotypes for the next generation of HPV tests for use in high-resource and low-resource settings. The implications of including possibly HPV genotypes on HPV test performance, also for guidance on the use of these tests for cervical cancer prevention programs, are discussed.

## Commentary

Periodically the International Agency for Research on Cancer (IARC) convenes a meeting of cancer experts to review and update the evidence regarding potential carcinogens. In February of 2009, the meeting for the 100^th ^IARC monograph convened on the carcinogenicity of biological agents, including human papillomavirus (HPV), now widely acknowledged as the obligate cause of cervical cancer and the important cause of other epithelial cancers, updating the evidence from the 90^th ^monograph meeting in 2005 [1,2]. One of the primary goals of this meeting was to examine the evidence for potential for each of the more than 100 HPV genotypes to cause cancer. The meeting report by Bouvard *et al. *[3] provides an overview of the meeting for all biological agents and highlights that cervical cancer is caused by HPV types that belong to a few phylogenetically related "high-risk" species (apha-5, 6, 7, 9 and 11) of the mucosotropic alpha genus. Schiffman *et al*. in the accompanying article provide the rationale that was used at the meeting to classify the mucosotropic HPV types as cancer-associated (carcinogenic) or not. Based on large case series, meta-analyses, and laboratory data of mechanistic studies, twelve HPV genotypes (HPV16, 18, 31, 33, 35, 39, 45, 51, 52, 56, 58, and 59) were deemed carcinogenic and HPV68 was considered probably carcinogenic.

One of the important outcomes of the meeting was the downgrading of HPV66 from the category of probably carcinogenic (Group 2A), as judged in 2005, to possibly carcinogenic (Group 2B), which also includes HPV genotypes HPV26, HPV53, HPV67, HPV70, HPV73, and HPV82. As explained by Schiffman *et al*. [4,5], these HPV genotypes had limited epidemiological evidence to cause cancer but are genetically related to other HPV genotypes that are carcinogenic. Unlike for HPV68, which is known to maintain a cancer cell line (ME 180), there are as yet only scarce laboratory data showing carcinogenic properties of few of the HPV genotypes classified as possibly carcinogenic (Group 2B) [3]. Furthermore, unfortunately, the available laboratory data did not provide information to understand the differences in carcinogenetic behavior between the HPV genotypes that belong to the "high-risk" alpha species [6,7].

Temporarily categorizing HPV66 as probably carcinogenic during the four-year interval meetings impacted the development of new clinical HPV assay. In 2005, there was only one FDA-approved HPV DNA test, hybrid capture 2 (hc2; Qiagen (formerly Digene), Gaithersburg, Maryland, USA), which targets the 13 certain and probably carcinogenic HPV genotypes as a pool of HPV genotypes. In 2009, one additional test, Cervista (Third Wave Technologies, Hologic, Inc., Madison, Wisconsin, USA), received FDA approval  and several other assays from other companies are being evaluated in clinical trials. Most have included HPV66 in their panel of targeted HPV genotypes.

What is the impact of including HPV66 in clinical tests? The lifecycle of these tests, from development to validation, is 10 years or more. As a consequence, there will be a generation of HPV tests (even some that are not now in clinical trials but too far along in the formulation phase to alter) that include HPV66 and in theory will be slightly less specific than what might otherwise be achieved. Thus, more women will test positive for HPV when used in primary screening or as triage/reflex test for equivocal (atypical squamous cells of undetermined significance [ASC-US]) cytology.

Worldwide, the prevalence of HPV66 estimated to be 0.4% (95%CI = 0.3–0.4%) although there are some regional variations in its prevalence [8]. Therefore, an additional 1 in 250 all women screened (400 in 100,000 women) will be called carcinogenic HPV positive because of the inclusion of HPV66. For simplicity, the contribution of HPV66 to testing positive has ignored the unknown fraction of HPV66 positives co-infected with carcinogenic and probable carcinogenic HPV genotypes. Of those who are HPV66 positive, perhaps another = 0.5% will have a concurrent diagnosis of cervical intraepithelial grade 2 (CIN2), equivocal precancer [9,10], or CIN3, more certain precancer, both of which are typically treated by excision or ablative procedures. Thus, the inclusion of HPV66 will result in the identification of HPV66-related CIN2/3 in roughly 2 in 100,000 women screened with the current HPV tests. Whether these HPV66-related CIN2/3 cases are clinically important, i.e. have invasive potential, or not remains unclear.

Future meetings will no doubt try to resolve whether these borderline carcinogenic HPV genotypes (HPV26, HPV53, HPV66, HPV67, HPV70, HPV73, and HPV82) are cancer causing or not. Integrative epidemiologic and further laboratory approaches will be needed for clarification. New investigative tools, such as micro-dissection of the lesion and HPV genotyping or *in situ *methods if they ever prove to be sufficiently sensitive, and genotype-specific detection of HPV E6/E7 transcripts or protein [11,12] may help to assign causal HPV genotypes.

In the end, perhaps resolving this issue of which of the borderline HPV genotypes are carcinogenic does not matter except for academic albeit important biological reasons: to understand the nature of HPV carcinogenicity and its relationship to viral genetics and phylogenetics [4]. We already know enough to decide which HPV genotypes to include in clinical HPV tests. As shown by others [5] using a receiver-operator curve (ROC)-like approach and CIN3 as the intermediate cancer endpoint, after including 8–10 most carcinogenic HPV genotypes there was only a slight increase in sensitivity and a larger cost in specificity with each additional HPV genotype included. However, CIN3 is subject to some diagnostic error [13,14], and some questionable or non-carcinogenic HPV genotypes can cause CIN3 that has little potential for invasion [14]. Thus, one would expect even greater clarity by redoing this analysis with a cancer endpoint using the accumulated data presented at the 100^th ^IARC monograph meeting. Experts then could come to a consensus about what is an acceptable tradeoff in sensitivity versus specificity vis-à-vis which HPV genotypes to include in clinical assays, accepting the notion that no test will achieve perfect sensitivity and that poor specificity has important implications, including potential unnecessary treatment, which can cause negative reproductive outcomes [15,16].

**Figure 1:**
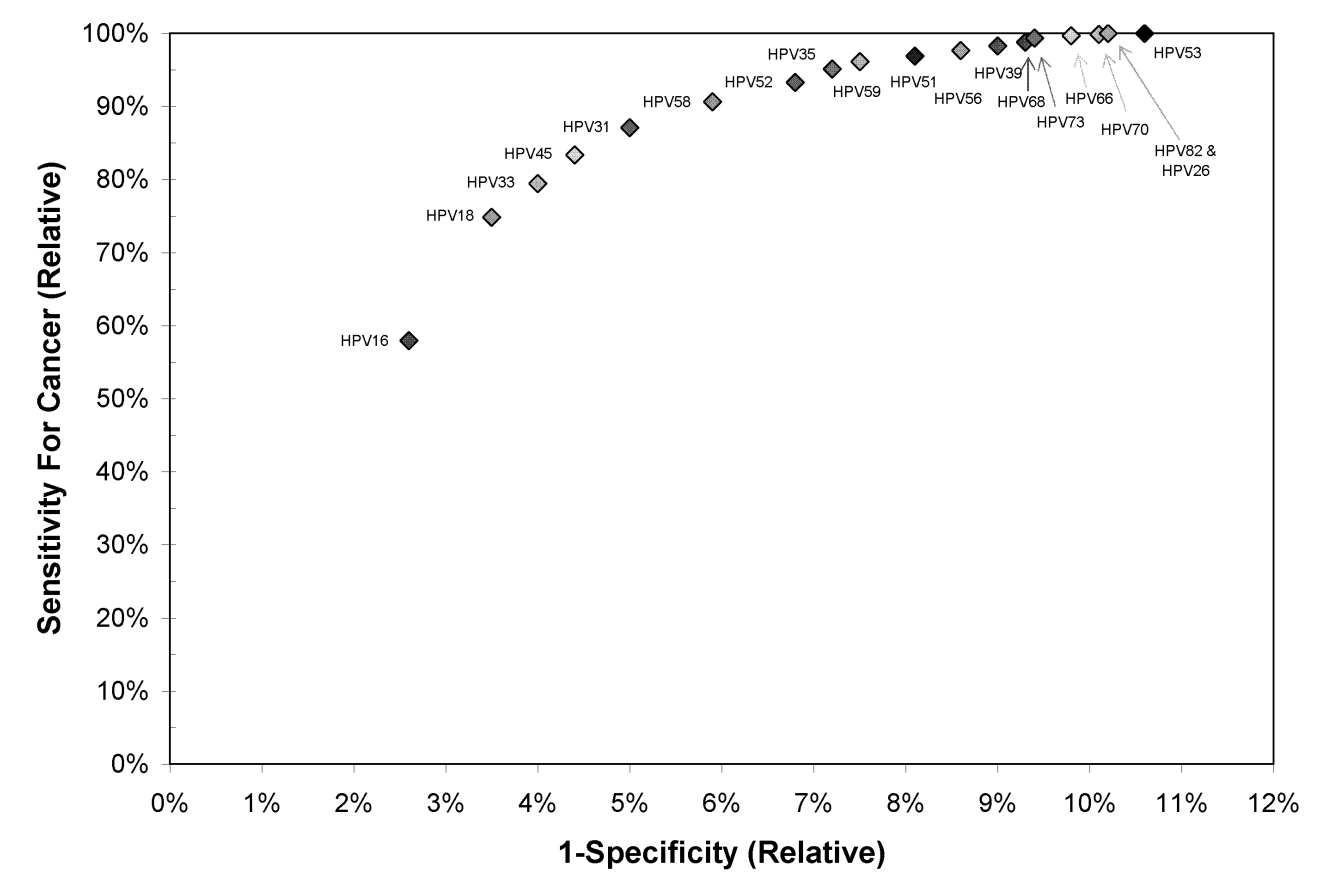
**The impact of adding individual carcinogenic, probable carcinogenic, and possible carcinogenic human papillomavirus (HPV) genotypes to a clinical HPV assay**. The impact of adding individual carcinogenic, probable carcinogenic, and possible carcinogenic human papillomavirus (HPV) genotypes to a clinical HPV assay was estimated using a receiver-operator curve (ROC)-like approach of stepwise adding individual HPV genotypes according to their prevalence, from most prevalent (HPV16) to least (HPV53), in cervical cancer [8,17]. Relative sensitivity (true positive fraction) was estimated based on those prevalences using the following assumptions: 1) HPV genotypes act independently such that cancers with multiple HPV genotypes detected were proportionally attributable to the HPV genotypes according to their overall prevalence; and 2) all cervical cancer is caused by HPV, and false negative results were independent of HPV genotype. Therefore, it was assumed that all cancers would have tested HPV positive with a perfectly sensitive test, and the relative contribution of each HPV genotype to cervical was a constant (although the absolute number increased). The relative false positive fraction, 1-Specificity, was estimated from the prevalence of individual HPV genotypes in the cytologic normal population, ignoring the overlap between HPV genotypes due to multi-HPV genotype infections (~25% of HPV positives).

As a first approximation, a simple version of this ROC-like analysis approach based on previously reported data [8,17] is shown in Figure 1. Notably, as previously noted [5], there is very little gain in sensitivity after the inclusion of the first ~10 HPV genotypes. Of note, the addition of the possible HPV genotypes decreases specificity by approximately 2%, or 2,000 additional HPV positives per 100,000 women, with virtually no benefit. Notably, most of these possible HPV genotypes (HPV53, HPV66, HPV67, HPV70, HPV73, and HPV82) are commonly detected by hc2 as the result of less than perfect targeting of the carcinogenic and possibly carcinogenic HPV genotypes [18]. Despite the cross-reactivity to these weakly carcinogenic or non-carcinogenic HPV genotypes, hc2 has been proposed as the current benchmark for HPV test performance [19,20]. More fidelity for the important HPV genotypes will improve the performance of HPV tests by reducing the number of false positive results. A more formal ROC-like analysis of the data from the 100^th ^IARC monograph [3] is needed to confirm these observations.

The inclusion or exclusion of borderline carcinogenic HPV genotypes in a clinical assay for carcinogenic HPV becomes moot if robust strategies for management (risk stratification) of test positives become available. That is, there would be no significant penalty to include some of these borderline HPV genotypes if there was downstream, specific method of triaging HPV-positive women to separate those in need of immediate colposcopy versus those who could be monitored by repeat screening in a year or two. The key is good stratification of risk for cervical precancer and cancer, and consistent, appropriate management of that risk [21]. Some approaches, such as using cytology [22], HPV genotyping [23], and/or p16 immunostaining [24], look promising but need further validation. New, as of yet undiscovered, biomarkers may provide still better solutions.

Although the inclusion of possibly carcinogenic HPV genotypes may hurt test specificity, it may indirectly increase the reassurance following a negative HPV test i.e. the negative predictive value of an HPV test for cervical precancer and cancer. Because non-carcinogenic and carcinogenic HPV infections alike are sexually transmitted, identification of women with borderline carcinogenic or non-carcinogenic HPV identifies a subset of higher-risk women who are more likely to subsequently acquire a new carcinogenic HPV infection. That is, women who are HPV positive for any HPV type at any given time point represent a sub-group that engages in more risky behavior, such as having more sexual partners, or have a partner(s) who is more likely to do so.

The future of cervical cancer screening in low-resource setting may include once-in-a-lifetime, low-cost and rapid HPV testing [25,26]. The inclusion/exclusion of HPV genotypes, and therefore the tradeoff of sensitivity versus specificity, will depend greatly on the strategy for following-up and managing the screen positives and the resources to do so. Prevention programs that will require the use of the more traditional screen, colposcopic evaluation of screen positives, and treatment of women with precancer will need sufficient numbers of highly-trained colposcopists to handle the clinical volume of screen positives, which could approach 10–15% of the population in many locations [27]. The tradeoff of more false positives for greater reassurance may not be an acceptable if the local infrastructure cannot manage the screen positives.

Other prevention programs may adopt screen and treat approaches, in which all screen positives undergo some treatment. For example, HPV-positive women could then be evaluated by visual inspection with acetic acid to decide the best treatment option: chemotherapy or palliative care for women with obvious cancer, surgical lesion excision (by a physician at a regional medical center) for large precancerous lesions that are not amenable to cryotherapy, and cryotherapy for all other HPV-positive women. Such an algorithm would only be applied to populations of older women, more than 10–15 years past the population median age of sexual debut, to minimize the over-treatment of mostly transient HPV infections in reproductive-age women. In general, because HPV-positive women will not undergo any further characterizations such as colposcopy or a secondary test, there should be a greater emphasis on specificity i.e. inclusion of only the certain carcinogenic HPV genotypes. However, there is some evidence to suggest that cryotherapy provides a secondary benefit of reducing the likelihood of acquiring new HPV infections post-treatment [28], which might increase the acceptability of over-treatment of screen positives when applied once to a population.

In the end, it seems unlikely that companies that develop HPV tests will tailor their tests to individual country needs. Now is the time for the community of scientists, doctors, and public health advocates to use the data presented at the 100^th ^IARC monograph meeting to rationally decide the target HPV genotypes for the next generation of HPV tests for use in high-resource and low-resource settings. And provide guidance on the use of these tests for cervical cancer prevention programs. However, the adoption of any cervical cancer screening and prevention program will depend on its social and cultural acceptance as well as available resources and the political willpower to implement it.

## References

[B1] (2007). Human Papillomaviruses. [90]. IARC Monographs on the Evaluation of Carcinogenic Risks to Humans.

[B2] Cogliano V, Baan R, Straif K, Grosse Y, Secretan B, El Ghissassi F (2005). Carcinogenicity of human papillomaviruses. Lancet Oncol.

[B3] Bouvard V, Baan R, Straif K, Grosse Y, Secretan B, El GF (2009). A review of human carcinogens – Part B: biological agents. Lancet Oncol.

[B4] Schiffman M, Herrero R, Desalle R, Hildesheim A, Wacholder S, Cecilia RA (2005). The carcinogenicity of human papillomavirus types reflects viral evolution. Virology.

[B5] Schiffman M, Khan MJ, Solomon D, Herrero R, Wacholder S, Hildesheim A (2005). A study of the impact of adding HPV types to cervical cancer screening and triage tests. J Natl Cancer Inst.

[B6] Hiller T, Poppelreuther S, Stubenrauch F, Iftner T (2006). Comparative analysis of 19 genital human papillomavirus types with regard to p53 degradation, immortalization, phylogeny, and epidemiologic risk classification. Cancer Epidemiol Biomarkers Prev.

[B7] Muench P, Hiller T, Probst S, Florea AM, Stubenrauch F, Iftner T (2009). Binding of PDZ proteins to HPV E6 proteins does neither correlate with epidemiological risk classification nor with the immortalization of foreskin keratinocytes. Virology.

[B8] de Sanjose S, Diaz M, Castellsague X, Clifford G, Bruni L, Munoz N (2007). Worldwide prevalence and genotype distribution of cervical human papillomavirus DNA in women with normal cytology: a meta-analysis. Lancet Infect Dis.

[B9] Castle PE, Stoler MH, Solomon D, Schiffman M (2007). The Relationship of Community Biopsy-Diagnosed Cervical Intraepithelial Neoplasia Grade 2 to the Quality Control Pathology-Reviewed Diagnoses:An ALTS Report. Am J Clin Pathol.

[B10] Castle PE, Schiffman M, Wheeler CM, Solomon D (2009). Evidence for Frequent Regression of Cervical Intraepithelial Neoplasia-Grade 2. Obstet Gynecol.

[B11] Spardy N, Duensing A, Charles D, Haines N, Nakahara T, Lambert PF (2007). The human papillomavirus type 16 E7 oncoprotein activates the Fanconi anemia (FA) pathway and causes accelerated chromosomal instability in FA cells. J Virol.

[B12] Wang HK, Duffy AA, Broker TR, Chow LT (2009). Robust production and passaging of infectious HPV in squamous epithelium of primary human keratinocytes. Genes Dev.

[B13] Stoler MH, Schiffman M (2001). Interobserver reproducibility of cervical cytologic and histologic interpretations: realistic estimates from the ASCUS-LSIL Triage Study. JAMA.

[B14] Castle PE, Cox JT, Jeronimo J, Solomon D, Wheeler CM, Gravitt PE (2008). An analysis of high-risk human papillomavirus DNA-negative cervical precancers in the ASCUS-LSIL Triage Study (ALTS). Obstet Gynecol.

[B15] Arbyn M, Kyrgiou M, Simoens C, Raifu AO, Koliopoulos G, Martin-Hirsch P (2008). Perinatal mortality and other severe adverse pregnancy outcomes associated with treatment of cervical intraepithelial neoplasia: meta-analysis. BMJ.

[B16] Kyrgiou M, Koliopoulos G, Martin-Hirsch P, Arbyn M, Prendiville W, Paraskevaidis E (2006). Obstetric outcomes after conservative treatment for intraepithelial or early invasive cervical lesions: systematic review and meta-analysis. Lancet.

[B17] Smith JS, Lindsay L, Hoots B, Keys J, Franceschi S, Winer R (2007). Human papillomavirus type distribution in invasive cervical cancer and high-grade cervical lesions: a meta-analysis update. Int J Cancer.

[B18] Castle PE, Solomon D, Wheeler CM, Gravitt PE, Wacholder S, Schiffman M (2008). Human papillomavirus genotype specificity of hybrid capture 2. J Clin Microbiol.

[B19] Stoler MH, Castle PE, Solomon D, Schiffman M (2007). The Expanded Use of HPV Testing in Gynecologic Practice per ASCCP-Guided Management Requires the Use of Well-Validated Assays. Am J Clin Pathol.

[B20] Meijer CJ, Berkhof J, Castle PE, Hesselink AT, Franco EL, Ronco G (2009). Guidelines for human papillomavirus DNA test requirements for primary cervical cancer screening in women 30 years and older. Int J Cancer.

[B21] Castle PE, Sideri M, Jeronimo J, Solomon D, Schiffman M (2008). Risk assessment to guide the prevention of cervical cancer. J Low Genit Tract Dis.

[B22] Naucler P, Ryd W, Tornberg S, Strand A, Wadell G, Elfgren K (2009). Efficacy of HPV DNA testing with cytology triage and/or repeat HPV DNA testing in primary cervical cancer screening. J Natl Cancer Inst.

[B23] Khan MJ, Castle PE, Lorincz AT, Wacholder S, Sherman M, Scott DR (2005). The elevated 10-year risk of cervical precancer and cancer in women with human papillomavirus (HPV) type 16 or 18 and the possible utility of type-specific HPV testing in clinical practice. J Natl Cancer Inst.

[B24] Carozzi F, Confortini M, Palma PD, Del MA, Gillio-Tos A, De ML (2008). Use of p16-INK4A overexpression to increase the specificity of human papillomavirus testing: a nested substudy of the NTCC randomised controlled trial. Lancet Oncol.

[B25] Qiao YL, Sellors JW, Eder PS, Bao YP, Lim JM, Zhao FH (2008). A new HPV-DNA test for cervical-cancer screening in developing regions: a cross-sectional study of clinical accuracy in rural China. Lancet Oncol.

[B26] Schiffman M, Wacholder S (2009). From India to the world – a better way to prevent cervical cancer. N Engl J Med.

[B27] Sankaranarayanan R, Nene BM, Shastri SS, Jayant K, Muwonge R, Budukh AM (2009). HPV screening for cervical cancer in rural India. N Engl J Med.

[B28] Wright T, Denny L, DeSousa M, Kuhn L (2009). Durable benefits of HPV-based screen-and-treat to 36 months. 25th International Papillomavirus Conference.

